# A Multidisciplinary Approach in the Management of Infectious Diarrhea in the Emergency Department

**DOI:** 10.7759/cureus.67788

**Published:** 2024-08-26

**Authors:** Muhammad Kalim Ullah, Fahad Dayam, Aamir Ahmed, Sohail Ahmad, Mehrub Munawar, Sidra Jahangir, Muhammad Humayun Daftani, Zeeshan Ali, Bakhtawar Kakar, Ammara Farooq, Naqeeb Ullah

**Affiliations:** 1 General Medicine, Rehman Medical Institute, Peshawar, PAK; 2 Internal Medicine, Mercy Teaching Hospital, Peshawar, PAK; 3 Community Health Sciences, Peshawar Medical College, Peshawar, PAK; 4 Internal Medicine, Lady Reading Hospital, Peshawar, PAK; 5 Anatomy, Jinnah Medical College, Peshawar, PAK; 6 General Practice, Burjeel Medical Hospital, Abu Dhabi, ARE; 7 Medicine and Surgery, Liaquat College of Medicine and Dentistry, Karachi, PAK; 8 Pulmonology, Lady Reading Hospital, Peshawar, PAK; 9 General Practice, Rahat Family Clinic, Peshawar, PAK

**Keywords:** infection control, patient outcomes, multidisciplinary approaches, emergency department, infectious diarrhea

## Abstract

Background

Globally, infectious diarrhea poses a serious threat to public health, especially in emergency department (ED) settings where timely and efficient treatment is essential. The primary objective of this study was to explore and evaluate the efficacy of a multidisciplinary approach (MDA) in the management of infectious diarrhea within the ED setting.

Methodology

This prospective cohort study was conducted from January to December 2023 at the Lady Reading Hospital ED in Peshawar, Pakistan. Individuals 18 years of age or older with a confirmed diagnosis of infectious diarrhea were included in the study. Those who refused to participate or had non-infectious reasons were excluded from the study. Through successive sampling, 650 patients comprised the study sample. Using a standardized form, demographic information, clinical presentations, test results, treatment methods, and patient outcomes were collected. Descriptive statistics and logistic regression were used in statistical studies to assess treatment outcomes and complications.

Results

Of the 650 patients, the majority (203 patients, 31.23%) were between the ages of 30 and 39 years. The gender distribution was about equal (332 men, 51.08%; 318 females, 48.92%). Abdominal pain (383 patients, 58.92%) was the most common presenting complaint. In 310 instances, bacterial pathogens were found, 160 had viral infections, and 110 had parasitic infections. There were notable complications, such as sepsis (37 patients, 5.69%), although the response to treatment effectiveness was notably high (593 patients, 91.08%). Upon follow-up, 590 (90.77%) patients had symptom and sign resolution, while 80 (12.31%) patients had a low recurrence rate. Longer symptom duration (odds ratio (OR) = 1.45, 95% confidence interval (CI) 1.12-1.88, p = 0.005) and specific antibiotics (OR = 1.68, 95% CI = 1.25-2.25, p = 0.001) were found to improve treatment success using logistic regression, whereas the risks of complications increased with age (OR = 1.05, 95% CI = 1.01-1.09, p = 0.012) and severe dehydration (OR = 1.75, 95% CI = 1.32-2.31, p < 0.001).

Conclusions

The MDA to manage infectious diarrhea in the ED significantly improved patient outcomes and reduced complications.

## Introduction

Globally, infectious diarrhea is a major public health concern, particularly in emergency department (ED) settings [1,2]. A multidisciplinary approach (MDA) that includes several medical disciplines, procedures, and healthcare strategies is necessary due to the complexity of this disorder [3]. Besides symptomatic treatment, infectious diarrhea care involves prompt diagnosis, suitable medication, infection control strategies, and patient education [4].

Effective coordination among healthcare personnel is critical in the ED, where some patients are brought with critical illnesses requiring prompt evaluation and management [5,6]. The seamless collaboration of emergency doctors, infectious disease experts, nurses, pharmacists, and laboratory technicians is crucial for providing precise diagnosis by prompt diagnostic testing and meticulous clinical evaluation [7]. This cooperative endeavor guarantees the timely implementation of suitable treatment plans customized to the particular pathogen detected, thereby lowering the complications and alleviating the strain on healthcare resources [8,9].

Furthermore, strict infection control procedures are needed to minimize nosocomial transmission in the care of infectious diarrhea in ED [1,10]. To restrict outbreaks and protect patients and healthcare staff, strict adherence to isolation procedures, hand hygiene practices, and environmental disinfection are essential elements of MDA infection control [11,12]. The dynamic nature of infectious diseases continually presents new challenges, emphasizing the ongoing need for innovative treatment strategies [13].

Even with advances in medical knowledge, there is still a significant study gap regarding the best way to include MDA for infectious diarrhea in ED settings. In particular, there is a paucity of thorough literature discussing the approach in which various specializations work in concert, the methods by which pathogens may be quickly identified, and the effects of teamwork on patient outcomes and healthcare costs. This study aims to bridge this gap by examining the integration of specific medical specializations, including infectious disease specialists, gastroenterologists, emergency physicians, and microbiologists. It will explore their roles in early diagnosis, appropriate management, and prevention strategies. Additionally, the study will assess the efficacy of quick diagnostic techniques and collaborative healthcare strategies to improve patient care outcomes in EDs managing infectious diarrhea cases.

Research objective

The primary objective of this study was to explore and evaluate the efficacy of MDA in the management of infectious diarrhea within the ED setting.

## Materials and methods

Study design and settings

This research was conducted from January 2023 to December 2023 at the Emergency Medicine Department of Lady Reading Hospital in Peshawar, Pakistan, utilizing a prospective cohort study design to evaluate MDA in treating infectious diarrhea.

Inclusion and exclusion criteria

Participants were required to be at least 18 years old and present with symptoms of infectious diarrhea, confirmed by laboratory tests. Clinical evaluation included assessing diarrhea, abdominal pain, fever, and exposure to infectious agents. Patients who were excluded included those who refused to participate, had non-infectious diarrhea (e.g., medication-induced), or had conditions requiring primary treatment outside the ED, such as severe sepsis or multi-organ involvement. This exclusion criterion was set to ensure the study focused specifically on those whose primary management occurred within the ED.

Sample size

During the research period, 650 eligible patients were consecutively sampled from the ED. Based on statistical considerations and the expected proportion of infectious diarrhea patients in the ED, this sample size was chosen to provide sufficient power for the study goals.

Data collection

A standardized data collection questionnaire created especially for this research was used to gather data. Demographic information; clinical presentation; laboratory results including stool cultures, parasite examinations, and polymerase chain reaction (PCR)-based stool tests for diagnosing infectious diarrhea; treatment modalities including antimicrobial therapy, rehydration with oral or intravenous fluids, and supportive care used; and patient outcomes during hospitalization and follow-up were among the data acquired.

Diagnostic tools and protocols

The MDA was clearly defined in this study as a comprehensive treatment protocol involving targeted antimicrobial therapy based on laboratory diagnostics, rehydration therapy (either oral or intravenous), and supportive care tailored to the patient’s clinical presentation. Diagnostic tools included stool cultures, parasitological examinations, and PCR tests to accurately identify the pathogens responsible for infectious diarrhea. The study adhered to strict protocols to ensure consistent application of the MDA. Treatment compliance was monitored through regular checks on medication administration and patient adherence to the rehydration regimen. Standard operating procedures were followed for each step of the MDA, from initial diagnosis to treatment and follow-up, ensuring that all participants received uniform care throughout the study.

Statistical analysis

Statistical analyses were conducted using SPSS version 25 (IBM Corp., Armonk, NY, USA). The research population’s demographic and clinical data were compiled using descriptive statistics, including means, frequencies, percentages, and standard deviations. Logistic regression analysis and other inferential statistics were used to assess relationships between variables and outcomes such as treatment success and side effects. Variables for logistic regression were selected based on their theoretical relevance and prior research suggesting their impact on outcomes, such as age, gender, severity of illness, and initial laboratory findings. This approach ensured a comprehensive analysis of predictors influencing treatment efficacy and adverse effects. Significance was determined at a p-value <0.05.

Ethical approval

The Institutional Review Board of Lady Reading Hospital in Peshawar, Pakistan, granted ethical clearance for this research before data collection began. Before their involvement in the study, all participants or their legal guardians provided informed consent, ensuring adherence to ethical standards of patient autonomy, beneficence, and confidentiality. For critically ill participants, consent was obtained from their legal guardians, with additional measures in place to ensure that the participants’ conditions and capacities were carefully considered throughout the process.

## Results

The demographic features of the study group that presented with infectious diarrhea in the ED are detailed in Table 1. The age distribution of the 650 patients were between the ages of 18 and 29 years (n = 147, 22.62%), 30 and 39 years (n = 203, 31.23%), 40 and 49 years (n = 179, 27.54%), 50 and 59 years (n = 92, 14.15%), and 60 years and older (n = 29, 4.46%). A total of 318 (48.92%) female patients and 332 (51.08%) male patients comprised the group. Regarding employment, 434 (66.77%) patients were employed, 81 (12.46%) patients were unemployed, 112 (17.23%) patients were students, and 23 (3.54%) patients fell into other groups. The study identified the following comorbidities: diabetes in 79 (12.15%) patients, hypertension in 41 (6.31%) patients, chronic kidney disease in 33 (5.08%) patients, and chronic liver disease in 29 (4.46%) patients.

**Table 1:** Demographic details of the study population.

Variable	Category	Number of patients (n)/Mean ± SD	Frequency (%)
Age (years)	18–29	147	22.62
	30–39	203	31.23
	40–49	179	27.54
	50–59	92	14.15
	60 and above	29	4.46
	Mean ± SD	35.25 ± 12.48	-
Gender	Male	332	51.08
	Female	318	48.92
Occupation	Student	112	17.23
	Employed	434	66.77
	Unemployed	81	12.46
	Other	23	3.54
Comorbidities	Diabetes	79	12.15
	Hypertension	41	6.31
	Chronic kidney disease	33	5.08
	Chronic liver disease	29	4.46

The study’s observations of clinical presentation features of patients with infectious diarrhea are included in Table 2. Clinical features were categorized by duration: <1 day (208 patients, 32.02%), 1-3 days (318 patients, 48.92%), and >3 days (124 patients, 19.08%). Among the patients, 412 (63.38%) were febrile while 238 (36.62%) were afebrile. Dehydration was classified as mild (281 patients, 43.23%), moderate (259 patients, 39.85%), severe (61 patients, 9.38%), or none (49 patients, 7.54%). Abdominal pain was reported by 383 (58.92%) patients, vomiting by 296 (45.54%) patients, and hematochezia by 182 (28.00%) patients.

**Table 2:** Clinical presentation of infectious diarrhea cases.

Variable	Category	Number of patients (n)	Frequency (%)
Duration of symptoms	<1 day	208	32.00
	1–3 days	318	48.92
	>3 days	124	19.08
Fever	Present	412	63.38
	Absent	238	36.62
Dehydration	Mild	281	43.23
	Moderate	259	39.85
	Severe	61	9.38
	None	49	7.54
Abdominal pain	Present	383	58.92
	Absent	267	41.08
Vomiting	Present	296	45.54
	Absent	354	54.46
Hematochezia	Present	182	28.00
	Absent	468	72.00

The laboratory results from the diagnostic tests performed on patients with infectious diarrhea are summarized in Table 3. In 310 patients, *Salmonella enterica* was the most common bacterial pathogen (18.31%, 57 patients), followed by *Shigella* (12.15%, 38 patients), *Campylobacter* (8.92%, 28 patients), and *Escherichia coli *(8.31%, 26 patients). Viral pathogens includedadenovirus(4.00%, 26 patients), rotavirus (8.92%, 58 patients), and norovirus (11.69%, 76 patients). Parasitic infections were predominantly *Giardia* (9.85%, 64 patients),* Cryptosporidium* (4.00%, 26 patients), and *Entamoeba histolytica* (3.08%, 20 patients). Mixed infections were found in 10.77% (70) of patients, with additional tests used in 7.69% of cases, and PCR and enzyme-linked immunosorbent assay (ELISA) in 27.69% and 18.46%, respectively.

**Table 3:** Laboratory findings in infectious diarrhea cases. ELISA: enzyme-linked immunosorbent assay; PCR: polymerase chain reaction

Diagnostic test	Pathogen species	Specific pathogen	Number of patients (n)	Frequency (%)
Stool culture	Bacterial (n = 310)	Salmonella enterica	119	18.31
		Shigella	79	12.15
		Campylobacter	58	8.92
		Escherichia coli (E. coli)	54	8.31
	Viral (n = 160)	Norovirus	76	11.69
		Rotavirus	58	8.92
		Adenovirus	26	4.00
	Parasitic (n = 110)	Giardia	64	9.85
		Cryptosporidium	26	4.00
		Entamoeba histolytica	20	3.08
	Mixed pathogens	Bacterial and parasitic pathogens	70	10.77
Other Tests	PCR	PCR tests	180	27.69
	ELISA	ELISA	120	18.46
	Other	*Giardia* antigen detection, *Cryptosporidium* antigen detection, and *Entamoeba histolytica* antigen detection	50	7.69

The research cohort’s treatment options for infectious diarrhea patients are listed in Table 4. The three categories of antibiotics prescribed were Type A (broad-spectrum, used empirically to target a wide range of bacteria), Type B (narrow-spectrum, selected based on specific pathogen identification), and Type C (reserved for resistant or less common pathogens, typically as second-line treatments). The distribution was 271 (41.69%) patients who received Type A, 225 (34.62%) patients who received Type B, and 154 (23.69%) patients who received Type C. Rehydration methods included oral for 393 (60.46%) patients, intravenous (IV) for 196 (30.15%) patients, and combined for 61 (9.38%) patients. Antiemetics were prescribed to 210 (32.31%) patients, while 440 (67.69%) patients did not receive them. Additionally, 120 (18.46%) patients received probiotics, 140 (21.54%) antidiarrheals, and 50 (7.69%) other specialized treatments.

**Table 4:** Treatment modalities employed in infectious diarrhea cases.

Treatment	Modality	Number of patients (n)	Frequency (%)
Antibiotics	Type A	271	41.69
	Type B	225	34.62
	Type C	154	23.69
Rehydration therapy	Oral	393	60.46
	Intravenous	196	30.15
	Combined	61	9.38
Antiemetics	Yes	210	32.31
	No	440	67.69
Other therapies	Probiotics	120	18.46
	Antidiarrheals	140	21.54
	Other (antisecretory agents and anti-inflammatory medications)	50	7.69

The study’s hospitalization outcomes for patients with infectious diarrhea are presented in Table 5. Of 650 patients, 593 (91.08%) improved clinically, while 57 (8.77%) did not. Complications included sepsis in 37 (5.69%) patients and other complications in 23 (3.54%) patients. No complications were reported for 590 (90.77%) patients. The average hospital stay was 4.5 days, with a standard deviation of 2.1 days.

**Table 5:** Patient outcomes during hospitalization.

Outcome	Variable	Number of patients (n)/Mean ± SD	Frequency (%)
Clinical improvement	Yes	593	91.08
	No	57	8.77
Complications	Sepsis	37	5.69
	Others (dehydration and electrolyte imbalances)	23	3.54
	None	590	90.77
Length of hospital stay (in days)	Mean ± SD	4.5 ± 2.1	

The results of patients with infectious diarrhea during follow-up after their hospital stay are shown in Table 6. Overall, 590 (90.77%) patients had a resolution, whereas 60 (9.23%) patients continued to have signs and symptoms such as abdominal cramps, diarrhea, nausea, vomiting, and fever. In total, 80 (12.31%) patients had a recurrence of diarrhea, whereas 570 (87.69%) patients did not. The average time that patients were followed up after being admitted to the hospital was 6.2 months, with a standard variation of 3.4 months.

**Table 6:** Patient outcomes at follow-up.

Outcome	Variable	Number of patients (n)/Mean ± SD	Frequency (%)
Resolution of symptoms	Yes	590	90.77
	No	60	9.23
Recurrence of diarrhea	Yes	80	12.31
	No	570	87.69
Follow-up duration		6.2 ± 3.4	-

The findings of logistic regression analysis analyzing the variables affecting treatment outcomes and complications in patients with infectious diarrhea are shown in Table 7. Age had a non-significant odds ratio (OR) of 0.98 per year increase (95% confidence interval (CI) = 0.95-1.01, p = 0.232) for treatment effectiveness. The success of therapy was not substantially impacted by gender (OR = 1.15, 95% CI = 0.89-1.48, p = 0.298). Conversely, the chances of treatment success were considerably higher for signs and symptoms that lasted longer (1-3 days vs. <1 day) (OR = 1.45, 95% CI = 1.12-1.88, p = 0.005). Fever had a negative correlation with the outcome of therapy (OR = 0.78, 95% CI = 0.61-0.99, p = 0.043). The presence of fever (OR = 1.31, 95% CI = 1.02-1.68, p = 0.036) and age (OR = 1.05 per year increase, 95% CI = 1.01-1.09, p = 0.012) were significant predictors for complications. Both chronic liver disease (OR = 1.59, 95% CI = 1.22-2.08, p = 0.001) and severe dehydration (OR = 1.75, 95% CI = 1.32-2.31, p < 0.001) substantially enhanced the risks of complications.

**Table 7:** Logistic regression analysis of variables associated with treatment success and complications. *: significant.

Variable	Outcome of interest	Odds ratio (OR)	95% confidence interval (CI)	P-value
Age (years)	Treatment success	0.98 (per year increase)	0.95-1.01	0.232
	Complications	1.05 (per year increase)	1.01-1.09	0.012*
Gender	Treatment success	1.15	0.89-1.48	0.298
	Complications	0.92	0.68-1.25	0.621
Duration	Treatment success	1.45 (1–3 days vs. <1 day)	1.12-1.88	0.005*
	Complications	1.22 (1–3 days vs. <1 day)	0.95-1.57	0.111
Fever	Treatment success	0.78	0.61-0.99	0.043*
	Complications	1.31	1.02-1.68	0.036*
Severity of dehydration	Treatment success	0.92 (moderate vs. mild)	0.68-1.25	0.641
	Complications	1.75 (severe vs. none)	1.32-2.31	<0.001*
Pathogen identified	Treatment success	0.95 (viral vs. bacterial)	0.71-1.27	0.734
	Complications	1.12 (parasitic vs. bacterial)	0.88-1.42	0.345
Use of antibiotics	Treatment success	1.68 (Type A vs. Type C)	1.25-2.25	0.001*
	Complications	1.29 (Type B vs. Type C)	0.99-1.68	0.056
Type of rehydration therapy	Treatment success	0.85 (IV vs. oral)	0.67-1.09	0.203
	Complications	1.12 (combined vs. oral)	0.88-1.42	0.347
Comorbidities	Treatment success	1.25 (diabetes vs. none)	0.97-1.60	0.084
	Complications	1.59 (chronic liver disease vs. none)	1.22-2.08	0.001

Table 8 details the MDA for treating infectious diarrhea, highlighting the roles of emergency medicine, infectious diseases, nursing, pharmacy, laboratory medicine, and clinicians or healthcare professionals. It underscores the importance of rehydration, antibiotics, infection control measures (e.g., isolation, hygiene, disinfection), and quick diagnostics (PCR, ELISA, stool cultures). Effective communication among healthcare providers is crucial for diagnosis, treatment, and patient education. This approach resulted in high treatment success (91.08%), low complication rates (5.69% sepsis), and positive follow-up outcomes (90.77% remission, 87.69% no recurrence).

**Table 8:** A multidisciplinary approach in the management of infectious diarrhea. ELISA: enzyme-linked immunosorbent assay; PCR: polymerase chain reaction

Component	Description
Medical specialties involved	Emergency Medicine, Infectious Disease, Nursing, Pharmacy, and Laboratory Medicine
Protocols and strategies	Rapid diagnostic tests (stool cultures, PCR, ELISA), treatment algorithms (antibiotics, rehydration therapy), and infection control measures (isolation protocols, hand hygiene, environmental disinfection)
Healthcare integration	Collaboration among healthcare professionals (physicians, nurses, technicians) for rapid diagnosis, timely treatment, and infection prevention
Patient education programs	Implementation of educational initiatives to educate patients on disease transmission, hygiene practices, and adherence to treatment
Patient outcomes	Enhanced treatment success rates (91.08%), reduction in complications (5.69% sepsis), and favorable follow-up outcomes (90.77% resolution, 87.69% no recurrence)

## Discussion

The purpose of the study was to assess the effectiveness of MDA in treating infectious diarrhea in the ED by examining clinical presentations, demographics, and treatment modalities. The results are in line with other studies that highlight the benefits of integrated healthcare practices and the significance of collaborative care in obtaining improved health outcomes [14].

The research population’s demographic data showed a balanced gender distribution (51.08% male, 48.92% female) and a wide age range, with the majority of participants falling between 30 and 39 years (31.23%) and 40 and 49 years (27.54%). This is consistent with a study reported by Guerrant et al. [15] that showed that although infectious diarrhea may afflict people of any age, it is more common in adults between the ages of 30 and 50 years because of lifestyle and occupational exposure such as healthcare workers and food handlers. Furthermore, the cohort’s comorbidities, which include diabetes (12.15%) and hypertension (6.31%), are consistent with a study by Kotloff et al. [16], suggesting that coexisting medical disorders may worsen the severity and complexity of diarrhea.

The clinical presentation was heterogeneous, with a considerable proportion of patients exhibiting fever (63.38%), signs and symptoms lasting 1-3 days (48.92%), and moderate-to-severe (9.38%) dehydration. These results are in line with those of Thielman and Guerrant [17], who highlighted the variation in the length and intensity of signs and symptoms in instances of infectious diarrhea. Notably, DuPont [18] indicated that the presence of hematochezia (28.00%) and vomiting (45.54%) highlights the necessity for quick diagnostic and therapeutic treatments.

According to laboratory results, 310 patients had bacterial infections, with *Shigella* (12.15%) and *Salmonella* (18.31%) being the most prevalent. There were 160 patients with viral pathogens, with norovirus (11.69%) accounting for the majority of the group. There were 110 cases of parasite infections, mostly *Giardia* (9.85%). These findings concur with the research of Kirk et al. [19], who found that identical pathogen prevalence was seen in diarrheal epidemics globally. The intricacy of infectious diarrhea is further highlighted by the mixed pathogens found (10.77%), which calls for thorough diagnostic methods, as suggested by Petri et al. [20].

Antibiotics were prescribed to 41.69% (Type A), 34.62% (Type B), and 23.69% (Type C) of patients, among a variety of other treatment options. The management of dehydration was greatly aided by rehydration therapy, both oral (60.46%) and IV (30.15%), which is in line with World Health Organization guidelines [21] regarding rehydration as the main therapeutic approach. A complete therapeutic strategy is seen in the use of antiemetics (32.31%) and adjuvant therapy such as probiotics (18.46%) and antidiarrheals (21.54%), which are in accordance with the clinical recommendations of the Infectious Diseases Society of America [22].

At follow-up, 91.08% of patients had demonstrated clinical improvement, and 90.77% had disease resolution. Relatively low rates of complications such as sepsis (5.69%) show how successful MDA is in lowering serious outcomes. These findings are consistent with those of Checkley et al. [23], who found that MDA techniques improved the management of diarrheal illnesses.

Significant predictors of treatment effectiveness and complications were also identified by the logistic regression analysis, with the presence of fever (OR = 0.78) and prolonged signs and symptoms duration (OR = 1.45) impacting outcomes. Significant predictors of complications were chronic liver disease (OR = 1.59) and severe dehydration (OR = 1.75). These results support those of Fischer Walker et al. [24], who highlighted the significance of comorbidities and clinical severity on the prognosis of diarrheal illness.

A limitation of this study on MDA for managing infectious diarrhea in the ED includes the potential for selection bias due to its prospective cohort design, as it only included patients presenting to a single tertiary care center, Lady Reading Hospital in Peshawar, Pakistan, and lacked a control group. This limitation may affect the representation of diverse demographic and clinical profiles found in other healthcare settings. Moreover, the study’s reliance on data from a single institution restricts the generalizability of the findings to broader populations and different settings with varying healthcare resources and patient demographics. Consequently, while the study offers valuable insights, caution is warranted when applying its findings in the future.

## Conclusions

The implementation of a MDA in the management of infectious diarrhea within the ED has demonstrated substantial improvements in patient outcomes. This approach, which integrates rapid diagnostic techniques, tailored treatment protocols, and effective infection control measures, resulted in a high treatment success rate and a low complication rate. The study highlights that collaborative care involving emergency physicians, infectious disease specialists, nurses, and other healthcare professionals enhances diagnostic accuracy and therapeutic efficacy. Notably, prolonged illness duration and specific antibiotic use were associated with improved treatment outcomes, while age, fever, and severe dehydration were significant predictors of complications. These findings underscore the effectiveness of a coordinated, comprehensive treatment strategy in reducing the severity and recurrence of infectious diarrhea, thereby optimizing patient care and resource utilization in the ED setting.

## Appendices

**Table 9:** Clinical assessment questionnaire. ELISA: enzyme-linked immunosorbent assay; PCR: polymerase chain reaction

Section	Question	Options/Notes
Demographic information	Age	Enter the patient’s age in years
	Gender	Male, Female
	Occupation	Student, Employed, Unemployed, Other
	Comorbidities	Diabetes, Hypertension, Chronic Kidney Disease, Chronic Liver Disease, None
Clinical presentation	Duration of symptoms	< 1 day, 1-3 days, > 3 days
	Fever	Present, Absent
	Degree of dehydration	None, Mild, Moderate, Severe
	Abdominal pain	Present, Absent
	Vomiting	Present, Absent
	Blood in stool	Present, Absent
Laboratory findings	Stool culture	Salmonella, Shigella, Campylobacter, Escherichia coli (E. coli), None
	Viral pathogens	Norovirus, Rotavirus, Adenovirus, None
	Parasitic pathogens	Salmonella, Shigella, Campylobacter, Escherichia coli (E. coli), Norovirus, Rotavirus, Adenovirus, Giardia, Cryptosporidium, Entamoeba histolytica, Other, None
	Mixed pathogens	Present, Absent
Other diagnostic tests	Other diagnostic tests	PCR, ELISA, Other specific tests
Treatment modalities	Antibiotic type	Type A, Type B, Type C, None
	Rehydration therapy	Oral, IV, Combined, None
	Antiemetics	Yes, No
	Other therapies	Probiotics, Antidiarrheals, Other, None
Patient outcomes	Clinical Improvement During Hospitalization	Yes, No
	Complications during hospitalization	Sepsis, Other, None
	Length of hospital stay	Enter the number of days hospitalized.
Follow-up outcomes	Resolution of symptoms	Yes, No
	Recurrence of diarrhea	Yes, No
